# Non-surgical treatment of idiopathic gingival enlargement: A case report

**DOI:** 10.1097/MD.0000000000037448

**Published:** 2024-05-10

**Authors:** Mingjie Ge, Mengli Li, Liheng Shen

**Affiliations:** a Center for Plastic and Reconstructive Surgery, Department of Stomatology, Zhejiang Provincial People’s Hospital, Affiliated People’s Hospital, Hangzhou Medical College, Hangzhou, China.

**Keywords:** case report, gingival enlargement, non-surgical treatment, prognosis

## Abstract

**Background:**

Idiopathic gingival enlargement is associated with plaque, but other contributing factors are unclear. The prognosis of idiopathic gingival enlargement is closely related to the patient’s oral hygiene habits and regular follow-up.

**Case Presentation:**

This article reports a case of a 32-year-old male patient with idiopathic gingival enlargement. The patient presented to the department of stomatology with a 2-month history of gingival swelling and pain on the right upper posterior teeth. During the treatment, oral hygiene instruction, supragingival cleaning, subgingival scaling, and root planning were carried out, and part of the hyperplastic gingiva was taken and sent for pathology. Pathological examination showed gingival enlargement with chronic suppurative inflammation. At 4-month follow-up, the patient’s periodontal condition remained basically stable, and the gingival enlargement did not recur.

**Conclusion:**

The treatment of this case resulted in significant reduction of gingival swelling and patient’s pain reduction through non-surgical treatment and good plaque control, indicating that patients with idiopathic gingival enlargement can also achieve ideal results through non-surgical treatment. Through oral hygiene instruction, the patient mastered the method of self-plaque control, which is conducive to the long-term stabilization of the periodontal situation.

## 1. Introduction

Idiopathic gingival enlargement (GE) is defined as an abnormal overgrowth of gingival tissue. The etiology of GE is poorly understood, but may be attributed to plaque accumulation, systemic hormonal stimulation, anemia, medications, or idiopathic factors.^[1–6]^ Idiopathic GE is mainly characterized by localized GE that covers part of the tooth surface and, in severe cases, can displace and loosen teeth, affecting eating and aesthetics.^[1,3,7]^ Treatment of idiopathic GE usually includes non-surgical and surgical treatments.^[8,9]^ Non-surgical treatment is primarily concerned with reducing tissue inflammation through mechanical removal of plaque, and the key to successful treatment is effective basic periodontal therapy and good plaque control by the patient himself. In this article, we report a case of idiopathic GE in a 32-year-old man who was treated with nonsurgical therapy and plaque control with satisfactory results.

## 2. Case presentation

### 2.1. Chief complaints

A 32-year-old Chinese man presented to the department of stomatology with a 2-month history of gingival swelling and pain on his upper right posterior tooth.

### 2.2. History of present illness

Symptoms began 2 months prior to the visit with a swollen gingiva on the upper right posterior tooth that continued to worsen and become painful, interfering with eating and bleeding badly from brushing.

### 2.3. History of past illness

**Figure 1.**
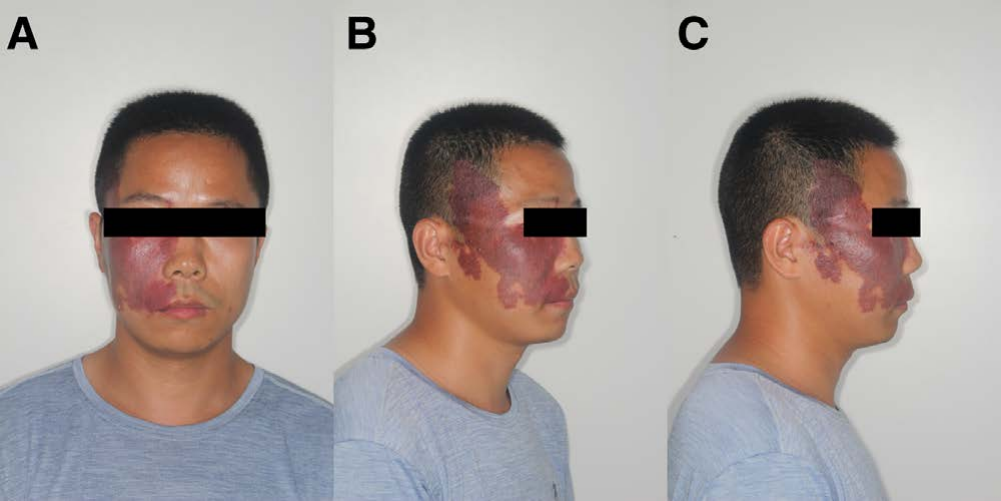
Photograph of the patient at the initial visit. (A) Frontal photograph; (B) 45° lateral photograph of the right side; and (C) 90° lateral photograph of the photo.

The patient denied to take antiepileptic drugs and cyclosporine. The patient has no history of hypertension, cardiovascular disease, denied a history of diabetes mellitus, epilepsy, and other systemic diseases. The patient was born with a grape-colored discoloration on the right side of the face, which gradually increased with growth and development and remained essentially unchanged in adulthood (Fig. 1). The patient denied surgery, trauma, blood transfusions, and a history of tetanus allergy.

### 2.4. Personal and family history

The patient denied any family history of gingival enlargement.

### 2.5. Physical examination

**Figure 2.**
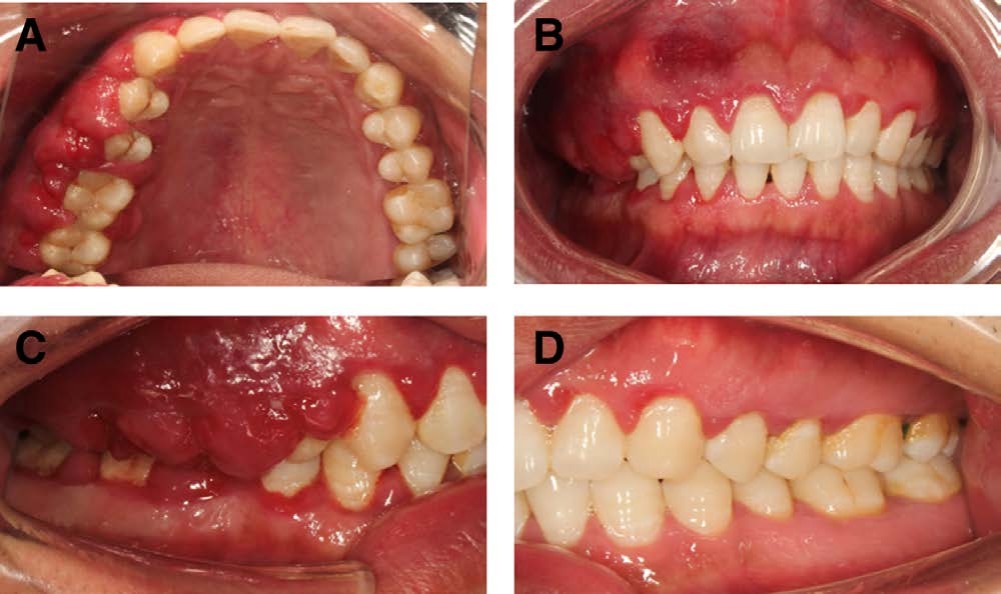
Gingival enlargement in the 32-year-old male patient without other diseases. In general, severe inflammation, redness of tissue, nodules, and calculus deposits were observed. (A) Upper occlusal view; (B) inter-occlusal view; (C) right side view; and (D) left side view.

**Figure 3.**
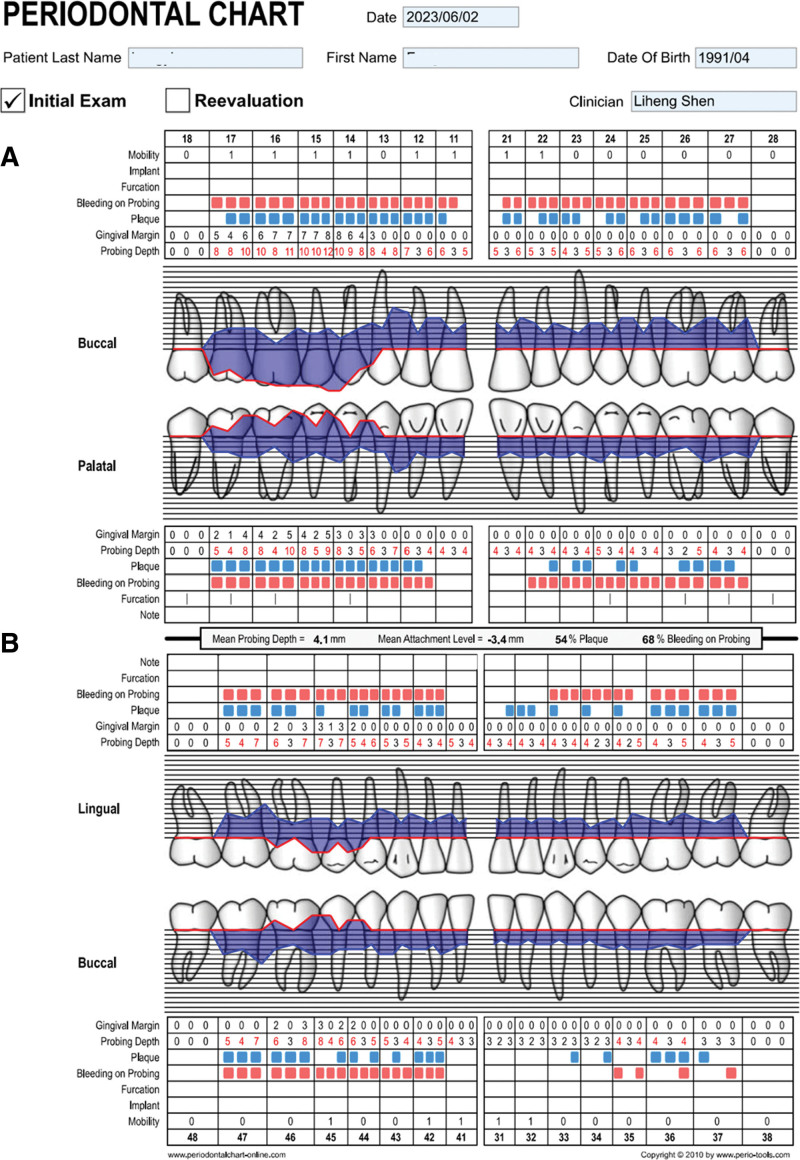
Periodontograms. (A) Upper and (B) lower.

On physical examination, the vital signs were as follows: Body temperature, 36.4°C; blood pressure, 113/71 mm Hg; heart rate, 79 beats per min; respiratory rate, 17 breaths per min. The patient’s face was symmetrical with no obvious defective deformity. The patient had flaky skin erythema on the right side of the face, irregular, well-defined, up to the forehead, down to the upper edge of the red lip, out to the front of the ear, and in to the nose, with thickening and no tenderness. There were no enlarged lymph nodes in the neck and submandibular region bilaterally. The patient’s aperture and opening pattern were normal, there was no popping and pain in the temporomandibular joints, and the mobility of the condyles on both sides was normal, with no compression pain. The teeth 13 to 17 buccal palatal gingival papillae showed bulbous enlargement, gingival papillae enlargement to about 10*3*5 mm^3^, soft texture, dark red color, spreading to attached gingiva, membranous gingival coalition, and covering 2/3 to all of the buccal and palatal surfaces of the teeth. The teeth 45 to 47 buccal lateral gingival papillae are bulbous with gingival papillae enlargement to about 5*3*4 mm^3^, loose texture, bright red color, spreading to the attached gingiva and covering about 1/3 of the buccal surfaces of the teeth (Fig. 2). The probing depth is about 5 to 10 mm, and the looseness of teeth 12 to 22, 31 to 42, and 45 is I degree (Fig. 3).

### 2.6. Laboratory examinations

No abnormality was found in routine blood, coagulation and liver and renal function tests.

### 2.7. Imaging examinations

**Figure 4.**
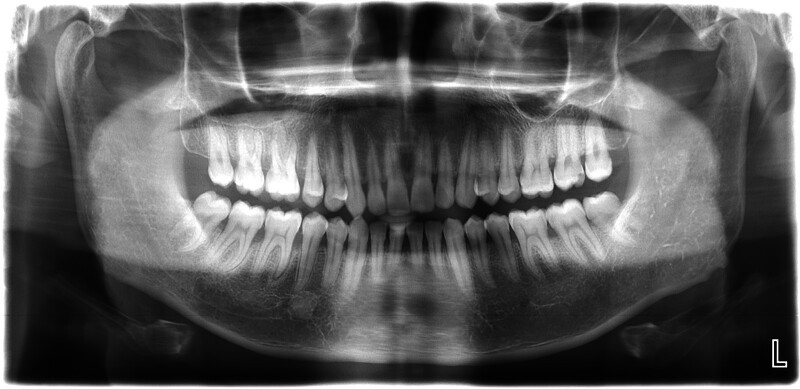
Panoramic X-Ray of the patient at the initial visit.

A panoramic photograph of the patient at the time of the initial consultation showed horizontal resorption of the alveolar bone throughout the mouth, with the alveolar bone of teeth 31, 36, 41, and 46 resorbing up to 1/3 of the length of the roots (Fig. 4).

## 3. Further diagnostic work-up

**Figure 5.**
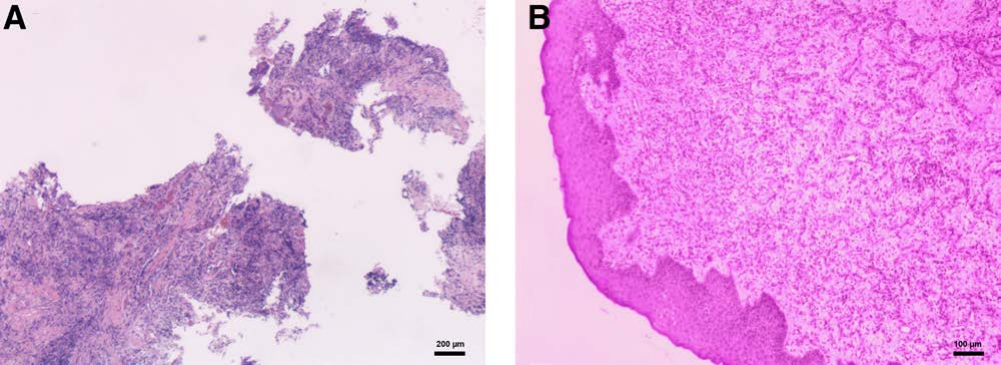
Histopathological analysis examination of the resected specimen. (A) Haematoxylin and eosin staining (×4). (B) Haematoxylin and eosin staining (×10).

The patient received biopsy and histopathological examination of the resected specimen (size, 0.5 cm × 0.4 cm × 0.3 cm) confirmed that the patient had gingival enlargement with chronic suppurative inflammation. The connective tissue showed large foci of chronic inflammatory infiltrate and lymphoplasmacyte diffusion in the collagen fibers and active fibroblast matrix (Fig. 5).

## 4. Final diagnosis

Based on all the information obtained from the clinical and radiographic examinations, the diagnosis was localized periodontitis stage I, grade B in teeth 31, 36, 41, and 46, and localized periodontitis. These were associated with a presumptive diagnosis of idiopathic gingival enlargement. The periodontitis was classified according to the new classification system of periodontal diseases and conditions of the American Academy of Periodontology and the European Federation of Periodontology.^[10]^

## 5. Treatment

**Figure 6.**
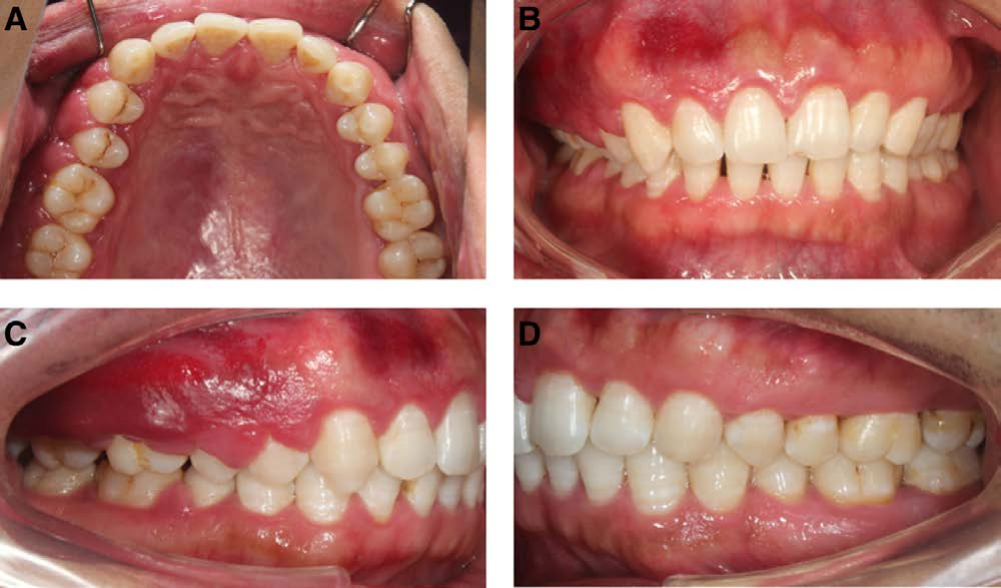
Patient with idiopathic gingival enlargement was reviewed 1 month after non-surgical treatment and the gingival swelling was significantly reduced. (A) Upper occlusal view; (B) inter-occlusal view; (C) right side view; and (D) left side view.

Before beginning of the treatment, a written consent was obtained from the patient. We treated the patient with non-surgical periodontal treatment. The patient underwent supragingival cleaning, subgingival scaling and root planning. The patient was given antibiotics for 3 days postoperatively, including cefuroxime sodium (0.25 g/dose, 2 times/day) and metronidazole (0.2 g/dose, 3 times/day). At the same time, we educated the patient about oral hygiene, which consisted of advising the patient on dental brushing, flossing, and rinsing with 0.12% chlorhexidine. One month after treatment, the patient’s gingival swelling of the right upper posterior tooth was significantly improved, pain was reduced, bleeding during brushing was significantly reduced, and he could use the affected side for chewing (Fig. 6).

## 6. Outcome and follow-up

**Figure 7.**
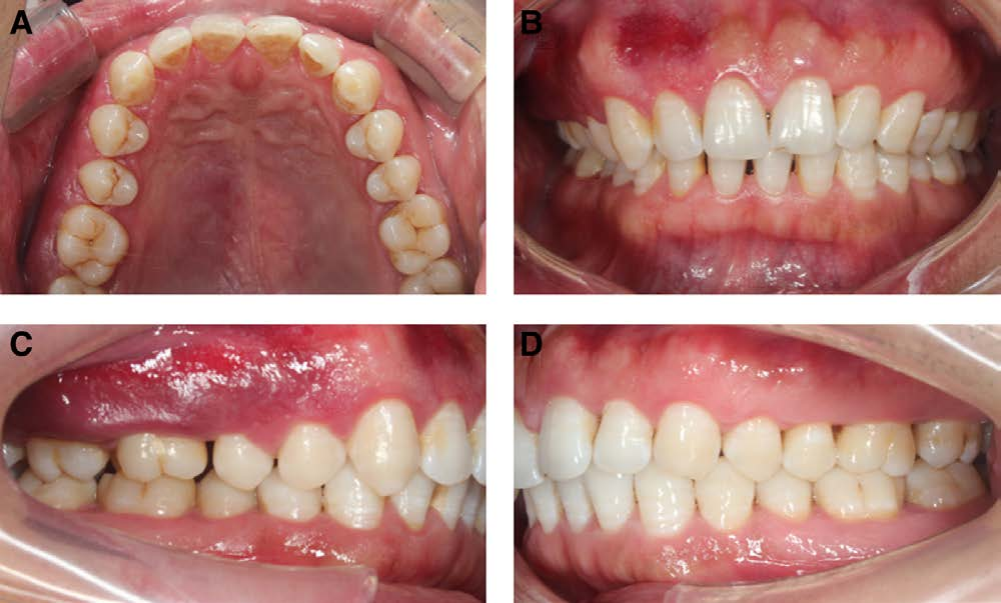
Patient with idiopathic gingival enlargement was reviewed 4 months after non-surgical treatment and the gingival swelling was significantly reduced. (A) Upper occlusal view; (B) inter-occlusal view; (C) right side view; and (D) left side view.

**Figure 8.**
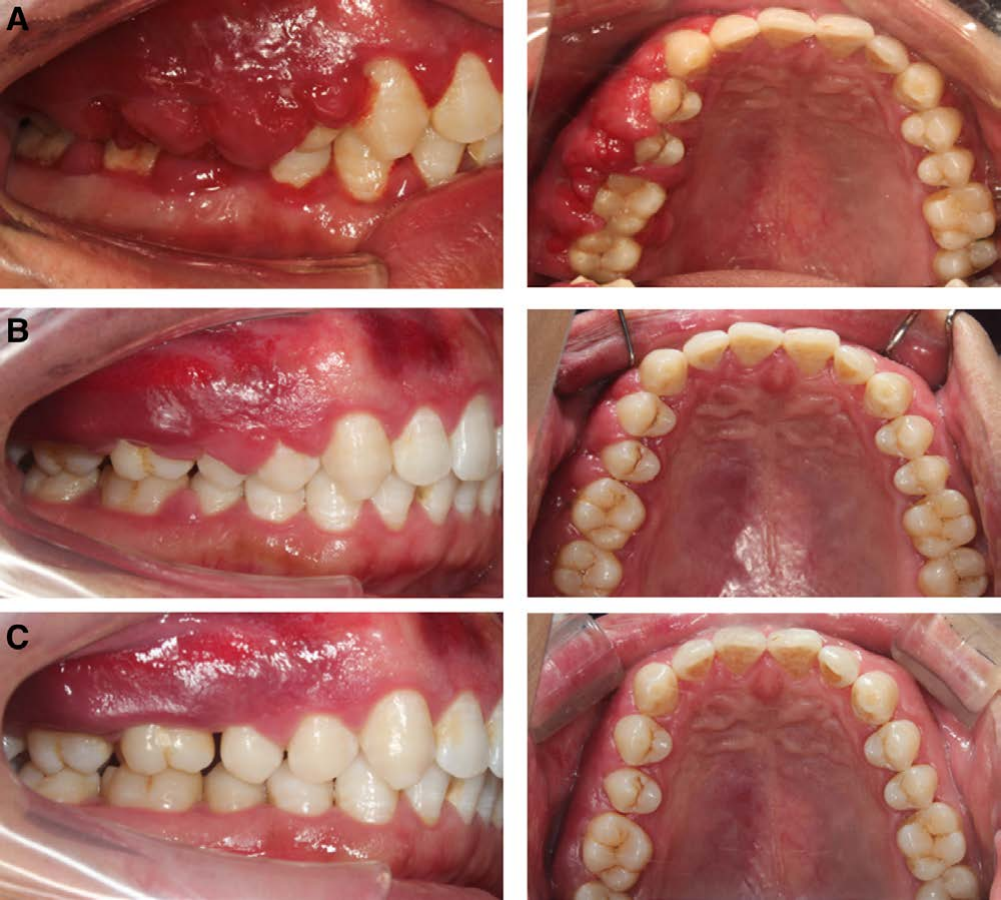
Patient with idiopathic gingival enlargement before and after treatment and follow-up. (A) Initial visit; (B) 1 mo after treatment; and (C) 4 mo after treatment.

The patient in this case had good compliance and a 4-month follow-up was performed. Four months after treatment, the patient’s periodontal condition remained stable, gingival enlargement did not recur and the patient was satisfied with the results of the treatment (Fig. 7). A comparison of his initial photographs with those taken at 1 month and 4 months after treatment reveals the changes brought about by periodontal treatment (Fig. 8).

## 7. Discussion

Gingival enlargement can occur alone or as part of a syndrome. Complications associated with gingival enlargement include tooth movement, poor plaque control, poor chewing, retention of milk teeth, delayed eruption of permanent teeth, aesthetics and malocclusion. Several common etiologies have been identified, including drug-induced, genetic, hormone-related (pregnancy), inflammatory, and systemic (leukemia). Depending on the etiology, gingival enlargement varies in clinical presentation, severity, development and duration. Based on the patient’s medical and dental history, it is difficult to establish a direct causal relationship. The patient denied systemic disease and denied taking medications, including cyclosporine, which causes GE.^[11]^ Therefore, drug-induced GE was ruled out. We ruled out hereditary gingival fibromatosis because there was no relevant family history.^[12]^

However, the patient had a congenital port-wine stain on the right side of the face (Fig. 1). Sturge-Weber syndrome (SWS) is a rare sporadic neurocutaneous syndrome characterized by bright red nevus on the face.^[13–15]^ It is caused by a somatic mosaic mutation in the GNAQ gene on chromosome 9q21 that affects neural crest cells in the forebrain region, resulting in vascular abnormalities in the prefrontal skin, cerebral cortex, and eyes.^[16]^ Patients with SWS usually have at least two of the following 3 components: Port wine stains on the face, cerebral vascular malformations, and ocular vascular malformations.^[17,18]^ In this case, the patient had a port-wine stain on the face at birth, and the patient refused further ophthalmologic examinations and cerebrovascular interventions, so the diagnosis of SWS could not be made for the time being. In the oral cavity, SWS manifested as a hemangiomatous lesion involving the mucosa.^[19]^ Based on the patient’s clinical examination, imaging, comparison of the healthy and affected sides of the gingiva, and general condition, we diagnosed the patient with idiopathic GE, which may be associated with facial capillary malformations.

Treatment of idiopathic gingival hypertrophy usually consists of both non-surgical and surgical treatments.^[8]^ The main point of non-surgical treatment is to mechanically remove plaque and thereby reduce tissue inflammation, and the key to success is effective periodontal basal therapy and the patient’s own good plaque control.^[20,21]^ It has been suggested that the observation period of periodontal basic therapy should be extended to provide more time for gingival tissues to recover.^[8,22]^ In our case, we were able to achieve a significant reduction in gingival enlargement with non-surgical treatment and good plaque control, and at 4 months follow-up, the patient’s periodontal condition was stable and the number of BOP-positive sites had decreased. However, 50% of patients with gingival hypertrophy recurred 15 months after periodontal treatment.^[7]^ Therefore, patients need to be instructed to maintain good oral hygiene and regular follow-up to prevent recurrence.

## 8. Conclusion

Treatment of idiopathic gingival enlargement usually includes both nonsurgical and surgical treatments, with nonsurgical treatments reducing gingival inflammation through strict plaque control. The observation period for basic periodontal treatment should be extended to give the gingival tissue more time to recover. The treatment of this case resulted in a significant reduction of gingival enlargement in the patient through nonsurgical treatment and good plaque control, suggesting that patients with idiopathic gingival enlargement can also achieve desirable results through nonsurgical treatment. Through oral hygiene instruction, the patient mastered the method of plaque self-control, which is conducive to long-term stability of the periodontal condition.

## Author contributions

**Conceptualization:** Mingjie Ge, Mengli Li, Liheng Shen.

**Data curation:** Mingjie Ge, Mengli Li, Liheng Shen.

**Formal analysis:** Mingjie Ge, Liheng Shen.

**Methodology:** Mengli Li, Liheng Shen.

**Project administration:** Mengli Li, Liheng Shen.

**Resources:** Liheng Shen.

**Software:** Mingjie Ge, Mengli Li.

**Supervision:** Liheng Shen.

**Validation:** Mengli Li.

**Visualization:** Mingjie Ge, Mengli Li.

**Writing – original draft:** Mingjie Ge, Mengli Li.

**Writing – review & editing:** Liheng Shen.
